# An Overview of Predictors for Intrinsically Disordered Proteins over 2010–2014

**DOI:** 10.3390/ijms161023446

**Published:** 2015-09-29

**Authors:** Jianzong Li, Yu Feng, Xiaoyun Wang, Jing Li, Wen Liu, Li Rong, Jinku Bao

**Affiliations:** 1 College of Life Sciences & Key Laboratory of Ministry of Education for Bio-Resources and Bio-Environment, Sichuan University, Chengdu 610064, China; E-Mails: lijianzong@hotmail.com (J.L.); dorisfeng2@sina.com (Y.F.); wxyun08109@163.com (X.W.); lijing_22@aliyun.com (J.L.); cindylwen@163.com (W.L.); rongronglili2014@163.com (L.R.); 2 State Key Laboratory of Biotherapy/Collaborative Innovation Center for Biotherapy, West China Hospital, Sichuan University, Chengdu 610041, China; 3 State Key Laboratory of Oral Diseases, West China College of Stomatology, Sichuan University, Chengdu 610041, China

**Keywords:** intrinsically disordered proteins, predictor, computational methods

## Abstract

The sequence-structure-function paradigm of proteins has been changed by the occurrence of intrinsically disordered proteins (IDPs). Benefiting from the structural disorder, IDPs are of particular importance in biological processes like regulation and signaling. IDPs are associated with human diseases, including cancer, cardiovascular disease, neurodegenerative diseases, amyloidoses, and several other maladies. IDPs attract a high level of interest and a substantial effort has been made to develop experimental and computational methods. So far, more than 70 prediction tools have been developed since 1997, within which 17 predictors were created in the last five years. Here, we presented an overview of IDPs predictors developed during 2010–2014. We analyzed the algorithms used for IDPs prediction by these tools and we also discussed the basic concept of various prediction methods for IDPs. The comparison of prediction performance among these tools is discussed as well.

## 1. Introduction

Intrinsically disordered proteins (IDPs) denote proteins (or regions/segments within proteins) characterized by the lack of stable secondary or tertiary structure under physiological conditions or in the absence of a binding partner [1]. Unlike structural (ordered) proteins that fold into a single, stable 3D structure, IDPs have no well-defined structure and exist as heterogeneous ensembles of rapidly interconverting conformations such that no single set of coordinates or backbone Ramachandran angles is sufficient to describe their conformational properties [2].

Since the lock-and-key mechanism recognized by Emil Fischer in 1894, the protein structure–function paradigm started ruling thoughts of researchers [3]. According to that, enzyme and substrate have to fit to each other like a lock and key in order to exert a chemical effect on each other. Later in the 1950s, the induced fit theory emerged, where enzymes are thought to be flexible when a substrate binds to the active site to make the reaction possible. This theory affected but did not change the sequence–structure–function concept. Then in 1978 when X-ray crystallography and NMR (nuclear magnetic resonance) successfully indicated functional disorder in proteins, the situation changed and research in IDPs developed and solidified. During the development of describing proteins or their regions that fail to form specific 3D structure, various terms were used, like floppy, pliable, rheomorphic, flexible, mobile, partially folded, vulnerable, chameleon, malleable, 4D, protein clouds, dancing proteins, proteins waiting for partners, and several other names often representing different combinations of “natively/naturally/inherently/intrinsically” with “unfolded/unstructured/disordered/denatured” [4]. At last, the term IDPs became more widely used than others in recent years.

IDPs carry out manifold functions as it is clear that structural disorder provides multiple functional advantages. Structural disorder is prevalent in proteins having regulatory roles but rare in enzymes, receptors, and proteins that require the precise spatial positioning of residues involved in ligand binding and catalysis. IDPs play important roles in determining the cell’s response to an external stimulus, transcription, translation, and assistance in the folding and unfolding of macromolecular structures in the cell [2]. IDPs can be classified into 28 separate functional categories. Alternatively based on their mode of action, they can be assigned to six broad classes comprising entropic chains, effectors, scavengers, assemblers, display sites, and chaperones [5].

Structural disorder is rather common in the higher eukaryotes. In humans, it is estimated that roughly one-third of all proteins is intrinsically disordered [6]. Approximately 50% of these proteins have stretches more than 30 residues long, and 25% are fully disordered [7]. Because of the fact that IDPs play crucial roles in numerous biological processes which mainly include recognition, regulation and cell signaling, it was not too surprising to find that some of them are involved in human diseases. Structural disorder was confirmed and studied in great detail in many other important disease-associated proteins, such as p53, T protein, and cystic fibrosis transmembrane conductance regulator [8]. IDPs are found to be involved in cancer, neurodegenerative diseases, cardiovascular diseases, diabetes, neural diseases, prion diseases, accelerated fibrillation, and protein deposition diseases [9].

Significant biological functions of IDPs boost researchers’ interests in this area, triggering rapid development of experimental and computational methods for prediction of disorder in proteins. Identification of IDPs is important because of the following reasons. Firstly, identifying disordered regions can promote protein analysis. Disordered regions in a protein have a biased amino acid composition that may give rise to inaccurate sequence alignments to unrelated proteins. By recognizing disordered regions, one can avoid aligning disordered regions with ordered regions and thus increase the accuracy of sequence similarity analysis [10]. Secondly, disordered regions often make the purification and crystallization of a protein difficult. The identification of a protein as highly disordered could save valuable time as researchers would not spend time attempting to determine a structure that does not exist [1]. Williams made the first attempt to predict lack of structure based on amino acid sequence as early in 1979 [11], but the first formal predictor was not published until 1997 [12]. After that, more than 50 predictors for IDPs prediction had been developed by 2009 [13]. From then onwards, 17 predictors for IDPs were created in the last five years. We reviewed those predictors here, for the sake of helping researchers select appropriate predictors to be used in their studies. In more detail we also show a practical example for the evaluation of their performance.

## 2. Predictors Developed in the Last Five Years

Predictors for IDPs prediction developed by 2009 had been well-discussed in He and colleagues’ review [13]. Seventeen new predictors were then created. These predictors can be roughly divided into three categories: (i) predictors based on machine learning classifiers; (ii) predictors based on a meta-approach which combines predictions from multiple predictors and (iii) predictors based on the physicochemical properties. Nevertheless, this classification is not absolute since some of the methods use more than one of these features, and combined web-based meta-servers also exist. The 17 predictors (Table 1) will be discussed in the following sections according to this classification.

**Table 1:** Description of Predictors for IDPs created during year 2010–2014.

Name	Year	PSI-BLAST	Availability
MFDp [14]	2010	X	X
PONDR-FIT [15]	2010	X	X
SPA [16]	2010		
DisCon [17]	2011	X	
POODLE-I [18]	2011		X
Cspritz [19]	2011	X	X
MetaDisorder [20]	2012	X	X
Espritz [21]	2012		X
SPINDE-D [22]	2012	X	
Dndisorder [23]	2013	X	X
IsUntruct [24]	2013		X
MFDp2 [25]	2013	X	X
RAPID [26]	2013		
PON-Diso [27]	2014	X	
DisMeta [28]	2014	X	X
DisPredict [29]	2014		
DISOPRED3 [30]	2014	X	X

Methods are sorted in ascending order by their year of publication. X represents the predictors that are publicly available and use the PSI-BLAST profiles.

### 2.1. Predictors Based on Machine Learning Classifiers

Many concepts have been put forward to predict IDPs. The prediction of protein disorder can be framed as a classic binary classification problem and targeted with various machine learning methods. To date, many machine learning algorithms for IDPs prediction have been published, including artificial neural network (ANN), support vector machine (SVM) and other homologous machine learning algorithms such as nearest neighbor algorithm, random forest Bayesian Markov chain models and so on.

ESpritz is based on bidirectional recursive neural network (BRNN) and trained on three different flavors of disorder [31,32]. The BRNN can be considered as an ensemble of three distinct neural networks, learning the C-terminal sequence context, the N-terminal sequence context and the general sequence respectively. This algorithm learns context information through the recursive dynamics of the network, reduces the number of parameters and extracts information implicitly from the sequence. ESpritz was trained by dataset from PDB (3244 proteins with 660,120 residues, of which 5.68% are disordered) and experimental data deposited in the Disprot database [33]. ESpritz consists of 20 inputs each unit is allocated for one of 20 amino acids. ESpritz is an efficient single sequence method, annotating entire genomes in the order of hours on a single processor core.

CSpritz is a web server for the prediction of intrinsic protein disorder [34]. This approach accomplishes the prediction through a combination of three machine learning systems including Spritz (based on PSI-BLSAT multiple sequence profiles and secondary structure), Punch (an SVM-based predictor extending Spritz), and ESpritz (an efficient BRNN based predictor). CSpritz not only uses those three approaches, but also takes into account the functional linear motifs [35,36] and secondary structure for disordered segments in to consideration for accurate prediction. CSpritz provides annotations about structural homologues and short functional linear motifs for disordered proteins. CSptritz focuses on elaborating single or multiple predictions for both short (≤30 residues) and long (>30 residues) disorder.

SPINE-D employs a single neural-network to predict disorder regions and is a new sequence based approach [37]. This novel method focuses on dealing with differences between long and short disorder regions. The features input by this method consist of predicted torsion angle fluctuations and predicted secondary structure [38]. For the input features, a 20-dimension PSSM vector is generated using three iterations of a PSI-BLAST search against the NCBI’s non-redundant protein sequence database. SPINE-D utilized the datasets from combinations of Disprot-annotated proteins and proteins directly from the PDB database annotated for disorder by missing coordinates in X-ray determined structures. This method provides simultaneous training for detecting short and long disordered regions. It aims to provide a tool that possesses the characteristic of consistently high accuracy in predicting both short and long disordered regions. And SPINE-D is one of the top servers according to the CASP9 ranking [39].

DNdisorder employs boosted ensembles of deep networks to predict disordered regions in proteins [40]. Deep networks (DNs) are similar to neural networks but contain more layers and are trained in a slightly different manner [41]. It is the first time to predict disorder proteins using such method. The dataset used for training is from 723 proteins originally used for the development of DISpro [42] and PreDisorder [43]. Those proteins are more than 30 residues in length, and comprised of 13909 disordered residues (about 6.5% disordered). In addition, DNdisorder also utilized the dataset from CASP9 and CASP10 [39,44], which are comprised of 117 and 95 proteins respectively. But the computational cost has been increased significantly because of using information derived from PSI-BLST.

DisPredict adopts a standard support vector machine that uses a radial basis function kernel and novel features for relative annotation of proteins [45]. Its dataset for training collected from a combination of protein sequences with disordered residues from both PDB and DisProt that is originally constructed for the development of MFDp [46]. Additionally, this method also utilized the dataset constructed for the development of SPINE-D. Benefitting from 10-fold cross validation and a more meaningful threshold for two-class classification of value 0.5, this method possesses features of higher accuracy and sensitivity, compared to MFDp and SPINE-D. DisPredict provides both the binary order/disorder assignment for each residue and the real value propensity of the disorder.

RAPID is a support vector regression-based predictor [47]. This method aims at providing a predictor that is fast speed, sophisticated design, and high-quality and robust predictive performance above batch (proteome-wide) predictions. This predictor is also designed and tested based on dataset originally created in MFDp and DisCon [48].

The series of DISOPRED are originally trained on evolutionarily conserved sequence features of disordered regions that have missing residues in high-resolution X-ray structures [49,50]. DISOPRED3 employs a novel SVM classifier to predict disordered regions and protein-binding sites [51]. Compared to DISOPRED2, by adding a nearest neighbor classifier, DISOPRED3 utilizes SVM and long-region neural network to predict disordered regions. Thus, the change is propitious to incorporate new data and DISOPRED3 is easy to update and maintain. As the continuation of the previous architecture of the series of DISOPRED, DISOPRED3 is shown to be more accurate than its predecessors [44].

There are tools capable of identifying disordered segments or disordered content. They also belong to the machine learning method. DisCon [48], PON-Diso [52], and SPA [16] are examples of such tools. DisCon uses a careful design of the information features and is designed to accurately predict the disorder content. DisCon performs the prediction of disorder content in three steps. Firstly, generating the positing specific scoring matrix (PSSM) and weighted observed percentage profiles using the PSI-BLST program [53]; Secondly, a set of numerical descriptors are generated using a series of relative prediction programs based on the PSSM profiles; Thirdly, a ridge regression model to generate predictions is built based on the small set of features. This method is designed and tested based on a dataset with 514 protein sequences that were collected from the PDB and the DisProt databases. This method aims to provide a high-quality alternative for high-throughput annotation and promote the binary annotations of disordered residues generated from other top-performance predictors. PON-Diso as a novel method is concerned with the disease mechanisms of amino acid substitutions and is developed to identify the effects of amino acid substitutions on protein disorder [52]. This method is based on the machine learning technique called random forest classifier [54]. This classifier is built on two sets of features, including features selected from AAindex and evolutionary sequence conservation [55]. SPA is a method that deals with disorder prediction in short peptides [16]. SPA utilizes two steps to predict disorder in short peptides. It first extends the inputted peptides by embedding it into a preselected segment of 30 residues. Then, it adopts PONDR VLXT [56] to analyze these extended peptides. PONDR VLXT uses a non-linear neural network classifier, trained to distinguish disordered/ordered based on coordination number, net charge, hydropathy, and the fraction of various amino acid groups.

### 2.2. Methods Based on a Meta-Approach Which Combines Predictions from Multiple Base Predictors

In this approach a prediction tool does not directly predict IDPs from the input information, instead it runs several IDPs prediction programs and makes a final combining prediction by taking into account all the results reported by a series of programs.

PONDR-FIT [57] is a meta-predictor, which combines six individual predictors including PONDR VLXT, VSL2, VL3 [58,59], FoldIndex [60], IUPred [61], and TopIDP [62]. The three predictors of PONDR series use artificial neural networks. FoldIndex, IUPred, and TopIDP to form disorder or ordered regions based on relative propensity of amino acids. The concerned six individual predictors emphasize different features of the sequence, and these methods are characterized by the high accuracy and reliability. PONDR-FIT integrates the results from six predictors using a single layer artificial neural network that is trained by eight-fold cross-validation. The analysis of accuracy indicates that PONDR-FIT improves the prediction accuracy with an average of 11% while compared to each of the six single predictors.

POODLE-I is a predictor based on the meta-approach [63]. It is a workflow system to predict disordered regions from the results of the series of POODLE programs. One of the advantages of POODLE-I over the other tools using meta-approach is that all the programs that are used as sub-modules in POODLE-I are in the same server. POODLE-I is designed by taking into account the detailed algorithm of each sub-module program.

MetaDisorder assembles 13 disordered predictors that perform well in CASP (critical assessment of structure prediction) experiments [64], including DisEMBL [65], DISOPRED2 [50], DISpro [42], Globplot [66], iPDA [67], IUPred, Pdisorder [68], Poodle-s [69], Poodle-L [70], PrDOS [71], Spritz [72], DisPSSMP [73], and RONN [74]. The results generated by these predictors are weighted by the accuracy of the methods. The MetaDisoder is not only a meta-predictor but also a web interface to a series of disorder meta-predictors. However, this meta-approach is quite slow due to consisting of numerous primary predictors.

DisMeta [75] is a meta-sever that has been developed by the NESG (Northeast Structural Genomics) consortium as a primary tool for design and optimization of protein constructs expressed for NMR and crystallization studies. DisMeta uses a consensus method that assembles a consensus result generated by eight primary sequence-based predictors including DISEMBL DISOPRED2, DISpro, FoldIndex, IUPred, RONN, and VSL2. This method also provides the analysis of secondary structure, signal peptides, transmembrane helical regions and low-complexity regions that is generated by PROFsec [76], SignalP [77], TMHMM [78], and SEG [79]. DisMeta has been used for protein construct design and optimization in the large-scale sample and structure production pipeline of the NESG consortium of the protein structure initiative. It is very successful in production of many protein samples that have been verified by experiment data.

MFDp [46] fuses three different methods that are complementary to each other, and utilizes a comprehensive selection of the input information sources. MFDp combines output from three SVMs with linear kernel and the resulting value is binarized using a threshold that equals 0.37. The combined orthogonal predictors include machine learning-based predictor DISOPRED2, IUPred that uses pairwise energy between amino acids to predict disorder and DISOclust [80] that is based on analysis of predicted 3D structural model. The three predictors are complementary to each other. Another expanded method named MFDp2 [81], which is also a meta-server that combines two methods including residues-level based MFDp and sequence-level based DISCon. Besides, MFDp2 is designed to be a user-friendly webserver.

### 2.3. Methods Based on the Physicochemical Properties

Propensity-based predictors like FoldIndex, GlobPlot, and FoldUnfold [82] rely on simple statistics of amino acid propensity, the physical/chemical features of amino acids, and a preliminary concept on the physical background of disorder. FoldUnfold is a well-known predictor using physical method. It detects protein disordered regions based on a parameter termed the mean packing density of residues. Another known physical method as IUPred was based on the same physical idea as FoldUnfold. There are rare methods based on the physical model in the past five years except IsUnstruct [83]. IsUnstruct employs the Ising model to distinguish disordered from the ordered regions based on statistic physics. This method performs well in predicting both short and long disordered regions and its accuracy is higher comparing to PONDR-FIT. In the framework of Ising model, each residue can be in one of the two states: ordered or disordered. The model is an approximation of the Ising model in which the interaction term between neighbors has been replaced with a penalty for changing between states (the energy of the border) [83].

## 3. A Brief Comparative Assessment of the Recent Developed Predictors of IDPs

In contents above, we briefly described IDPs predictors created during the past five years. To date, more than 70 predictors for disorder prediction have been developed. Those computational methods are capable of producing high throughput predicted annotation of disordered residues in IDPs, providing a reasonable solution to fill up the time consuming and costly experimental annotation gap with the rapid growth rate of known protein sequences.

Generally, most methods are based on the machine learning techniques. These methods have been widely used to predict protein disorder and they perform excellently in predicting IDPs [13,84]. But they are usually short of explanations for the underlying mechanisms due to their black-box nature. Alternatively, the biophysical methods for predicting IDPs average the physic-chemical properties over the sequences to derive a state-index to predict order or disorder. A statistical analysis of known ordered and disordered proteins allows for the creation of disorder propensities that can be used to predict disorder [61,85]. These approaches are fast and simple but do not utilize the data in an optimized way. These methods are usually not as accurate as the machine-learning-based methods, while the advantages of this approach are simpler (making them faster) and have a clearer meaning. Meta-predictors are a combination of aforementioned methods and are constructed by combining several predictors. This can be done by a simple average of output from each method or in a performance weighted manner. Taking into account different false positive rates of individual predictors, a performance weighted manner was more widely used than the former. It is not a bad idea to avert using one single algorithm when predicting disorder, which usually results in an improvement in performance. At CASP, the best predictors are widely known to be meta-predictors combining orthogonal information [39,44]. Generally, tools based on the physicochemical properties of disordered regions are faster than machine learning based methods which employ the components from PSI-BLAST or meta-approach based approaches.

Several pieces of overview work on the disorder prediction were published in the last couple of years [10,86,87,88]. Xin Deng *et al.*, present an overview of 23 disorder predictors on a benchmark of CASP 9 in 2011, and their work indicated no single perfect predictors [10]. Peng *et al.*, evaluated disorder predictions at the residues, segment, chain levels, and employed different evaluation criteria to estimate the considered predictors [86]. A number of currently popular disorder predictors were evaluated using a benchmark dataset that contains the CASP-like and the Disprot disorder annotations. Some predictors such as MFDp, VSL2B [89], and POLNDR-FIT tend to be excellent when using AUC (area under the ROC curve) measure. However, once the evaluation criteria change, the result will change. Lately, Ian Walsh *et al.*, adopted a new evaluation criterion that integrated 12 quality measures to conduct the first large-scale assessment of 11 predictors based on UniProt sequences [87]. The test dataset covers 25,833 UniProt sequences with disorder annotations from X-ray crystallographic structures. It also suggests a strong variability in predictors across the 12 measures due to different prediction styles. Moreover, Z. Dosztányi *et al.*, presented a small survey of current methods to identify disorder and they also discussed prediction performance of specific disordered regions such as binding domain [88].

Indeed, the old methods perform the predictions in a high-throughput manner and consequently they can be used as a possible solution to narrow the annotation gap. Since 2002, the disorder predictors are biannually assessed and compared during the critical assessment of structure prediction (CASP) experiments. Although they can achieve AUC of about 0.8 [28,40] and MCC (Matthews Correlation Coefficient) of about 0.45 [28]. Some studies suggest that they typically make relatively substantial mistakes. These methods may over-or under-predict the overall amount of disorder in the sequence. A benchmark test of 10 recent predictors shows that the average mean absolute errors between the native and the predicted amount of disorder per chain varied between 15% and 39% [30]. In another benchmark of 19 predictors the average mean absolute errors ranged between 15% and 44% [70]. One explanation for this is that most of these methods use a local/sliding sequence window to predict the disorder. Demand for improved predictors motivates research toward the development of computational methods that predict disordered regions more accurately.

**Figure 1:**
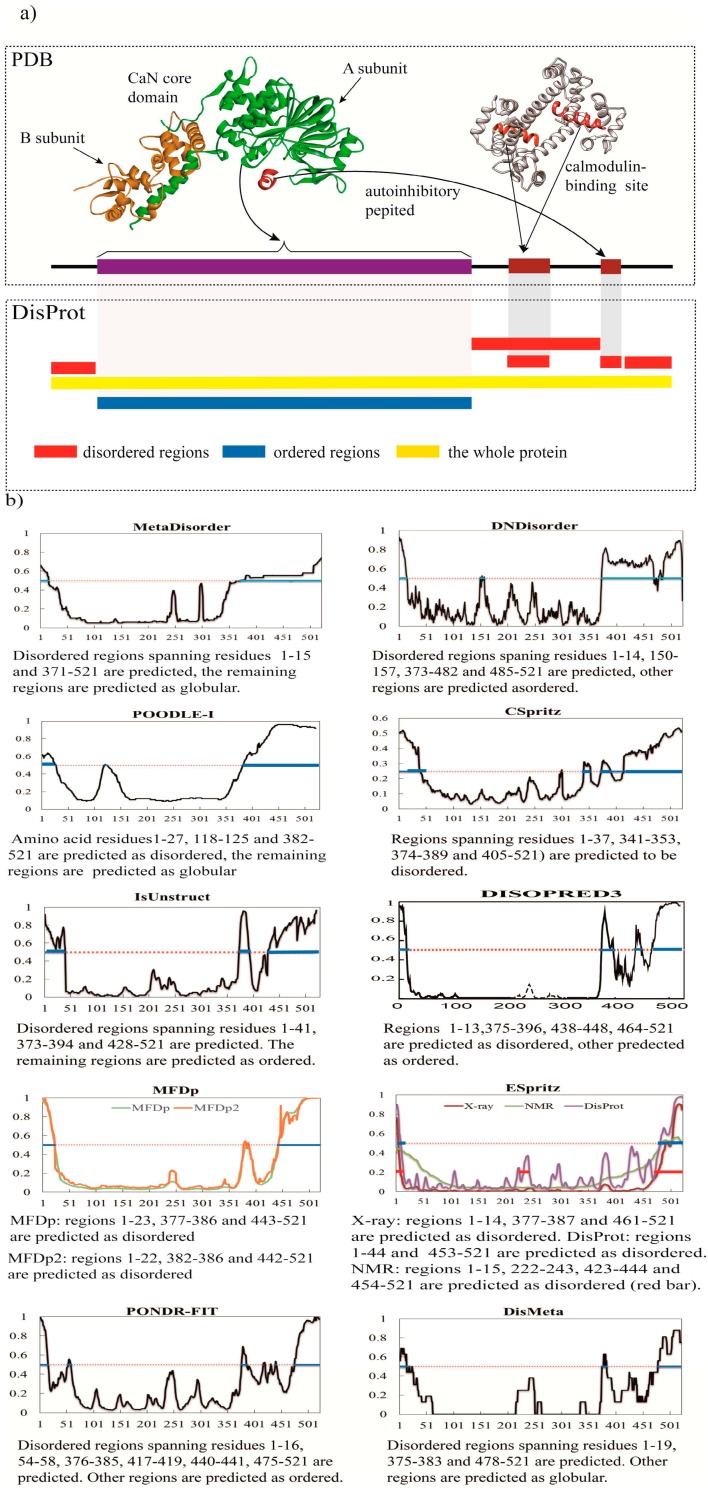

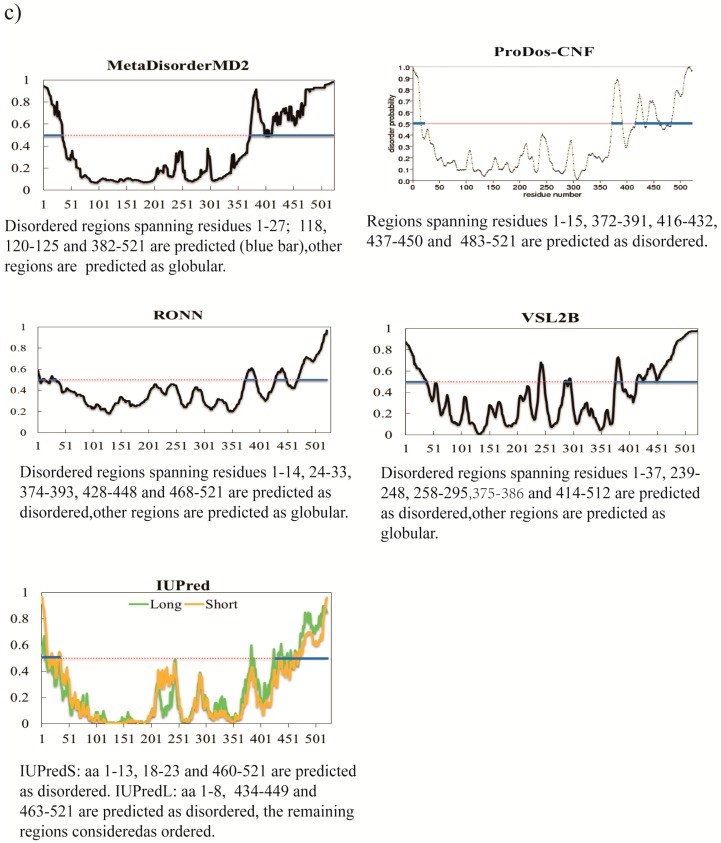
Analysis of calcineurin using different predictors. (**a**) Structure of calcineurin with essential disorder. Calcineurin (PDB 1AUI, top left) is composed of a catalytic A subunit (green) and a regulatory B subunit (saffron). Calcineurin also has an autoinhibitory peptide (dark red) and a calmodulin-binding site (red) located within the disordered region that becomes ordered upon binding (PDB 2R28, top right). The bottom plot shows annotations for regions according to PDB and DisProt database respectively; (**b**) Graphical output from 11 new disorder predictors; (**c**) Graphical output of five older top performance predictors, PrDos-CNF, MetaDisorderMD2, VSL2B, RONN, IUPred (short and long). In (**b**,**c**), red dot line is threshold, above which the region is disordered (blue line) and under which the region is ordered (red dot line), and the corresponding interpretation is shown under the graph as well.

To analyze the performance of predictors emerged over the past 5 years, we have chosen the human calcineurin (PDB code 1AUI) to test all the available predictors and we have also tested the older top performance predictors (Figure 1a–c). Biophysical and biochemical analyses have shown that this protein consists of a highly flexible region for CaM-domain [90]. It is a serine/threonine protein phosphatase consisting of a catalytic subunit A (DisProt accession: DP00092, Figure 1a) and a regulatory subunit B. The disordered region spanning residues 374–486 includes a CaM-binding domain lay between residues 390–414 that becomes ordered upon binding (shown in Figure 1a, PDB code 2R28). And on the N- and C-terminal, regions 1–13 and 48–521 are considered as disordered because its invisibility in the electron density map of the crystal structure. The predictors exploited include most of the available predictors described above and we also included some older top performance predictors, including PrDos-CNF (CASP10 wining method) [26], MetaDisorderMD2 (CASP9 wining method) [21], VSL2B (CASP9 wining method), RONN and IUPred series. The graphical output of each method and the corresponding interpretation are shown in Figure 1b,c. The overview statistic is shown in Table 2. In this table we use the accuracy value (ACC) to quantify the predictors’ performance. ACC = (TP + TN)/(TP + FP + TN + FN), where TP is the number of true positives (correctly predicted disordered residues), FP denotes false positives (structured residues that were predicted as disordered), TN denotes true negatives (correctly predicted structured residues), and FN denotes false negatives (disordered residues that were predicted as structured).

As shown in Figure 1b,c, for most regions, including the ordered catalytic domain and general disordered regions in the N- and the C-terminal regions (N terminal region 1–13 and C terminal region 487–521), the results reached a consensus among the methods. In this case, DisMeta and DISOPRED3 can precisely predict those regions as disordered, and have high accuracy. PONDR-FIT identifies the first 13 disordered residues perfectly. DNdisorder, PrDos-CNF, MetaDisoderMD2, and IUPredS (Figure 1c) also tend to respond to those regions uniformly. This situation is also adapted to the generally disordered segment that lies between the CaNB-binding helix (regions 343–373) and the CaM-binding domain (regions 390–414). Figure 1b,c illustrates a high consistency in recognizing generally disordered regions. The reason is that they have been trained on disorder data from both short regions of missing density from the PDB and experimental data as deposited in the DisProt (e.g., CSpritz, ESpritz, DNdisorder, PONDR-FIT and ESpritz).

**Table 2:** Overview statistic on different predictors.

Predictor	No. of Disordered Residues	Total % Disorder	ACC
Metadisorder	166	0.32	0.9804
Dndisorder	160	0.31	0.9731
MetaDisorderMD2	184	0.35	0.9559
POODLE-I	172	0.33	0.9539
Cspritz	173	0.33	0.9001
IsUnstruct	158	0.30	0.8848
PrDos-CNF	106	0.20	0.8752
RONN	115	0.22	0.8695
Espritz_NMR	144	0.28	0.8694
DISOPRED3	102	0.20	0.8675
MFDp	112	0.21	0.8656
MFDp2	107	0.21	0.8618
VSL2B	178	0.34	0.8580
Espritz_X-ray	85	0.16	0.8522
IUPredL	83	0.16	0.8503
IUPredS	81	0.16	0.8234
PONDR-FIT	84	0.16	0.8215
DisMeta	69	0.13	0.8157
Espritz_DisProt	113	0.22	0.7888

Methods are sorted in descending order by accuracy value.

Interestingly, most of the prediction scores are homogenous along the generic disordered regions. However, a typical situation in which predictors may disagree involves disordered regions that fold upon binding. Actually, only three new predictors predicted the CaM-binding (390–414) domain as disordered region accurately, including DNdisorder, Metadisorder, and POODLE-I. Their ACC values are also higher than other predictors (Table 2). Most of them belong to meta-method except DNdisordered, which is a machine learning predictor. The remaining predictors predicted these regions as ordered. Figure 1c illustrates several older well-performed approaches used to analyze the disordered regions of calcineurin. In contrast to other methods, the results obtained with RONN, IUPredS, and IUPredL do not identify these above-mentioned regions properly, indicating that, due to the uneven structural propensities of the protein, the predictions are not homogenous either. PrDos-CNF predicted this region as completely ordered, but the MetaDisorderedMD2, a meta-predictor, predicted regions spanning residues 382–521 as disordered. In contrast, some of the other predictors regarded as well-performing methods, such as VSL2B, failed to predict the disordered region correctly.

No predictors are found to be absolutely correct in the prediction of disorder. It should be stressed that it is difficult and maybe impractical to establish a perfect predictor at this moment. Moreover, depending on different predictors, the regions such as disordered binding region, coiled-coil, and molten globule can be predicted as completely disordered, completely ordered, or as borderline cases. Different predictors should be combined, as performed by meta-predictors, which seek a consensus of the scores of different predictors relying on different concepts.

## 4. Concluding Remarks

The enthusiasm for IDPs prediction grows increasingly. As mentioned above, the number of representative predictors has risen by 17 in the last five years. The process of designing and using these predictors benefits the discovery of significant biological and biomedical information. Even though the methodology of IDPs prediction has been improved dramatically, research in this field is still undergoing many difficulties. In fact, the available data are insufficient to create reliable computational tools. The databank of IDPs, DisProt, only includes 694 IDPs and 1539 disordered regions in the DisProt Release 6.02 (24 May 2013). This shortcoming indicates there is still an enormous gap between the number of annotated IDPs and the number of IDPs in nature, limiting the development of new predictors as well as the ability to improve already existing predictor algorithms.

As IDPs often have a close relation with some diseases, accurate IDPs prediction is getting more and more attention. In our opinion, future development of predictors predicting IDPs should be oriented towards the following aspects: (1) figuring out the molecular mechanisms of disordered structure formation clearly; (2) instead of using black-box computational techniques like SVM and ANN, prediction models integrating biophysical features of IDPs should be created; (3) improving the accuracy of prediction for IDPs by reducing the high noise level regarding both the structured and disordered regions that are used as training sets. Hopefully, prediction techniques are expected to continue to be very important for helping to develop an understanding of IDPs. With the continued development of prediction techniques, better predictors for IDPs are expected to be developed in the future.
